# Supplementary material for: Conditional chemoconnectomics (cCCTomics) as a strategy for efficient and conditional targeting of chemical transmission
Source: eLife. 2024 Apr 30;12:RP91927. doi: 10.7554/eLife.91927 (PMC11060718; doi:10.7554/eLife.91927)
Supplement: Figure 3—source data 3. [file elife-91927-fig3-data3.zip › Figure 3 source data3 part 1.zip!/Figure 3-Source data 3-part1/1071f3-1-CRISPResso_Report_jvi77nmx.zip!/CRISPResso_on_1071f3_3/9.Alleles_frequency_table_around_sgRNA_actactacatctccgtggag.pdf]

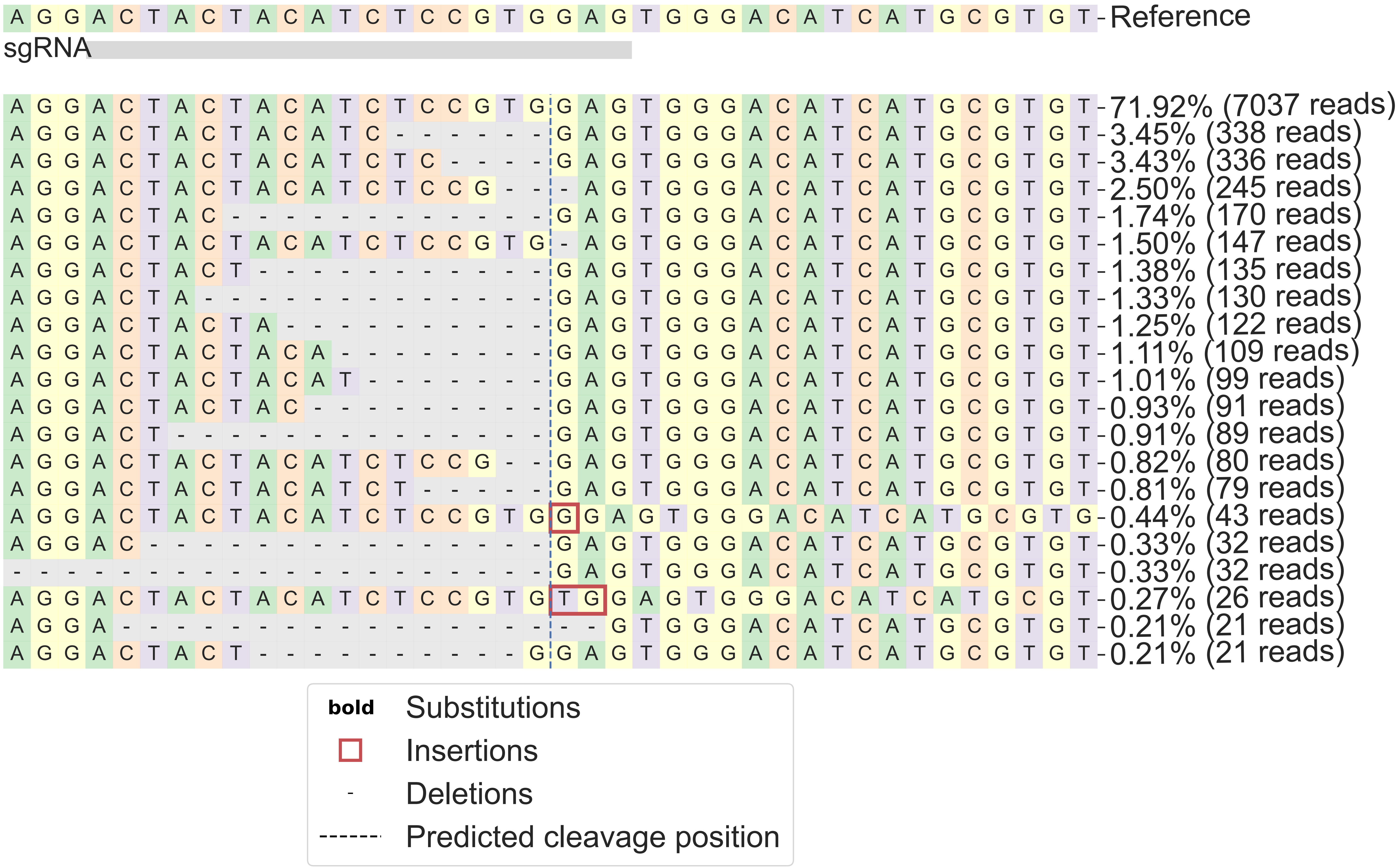
AGGACTACTACATCTCCGTGGAGTGGGACATCATGCGTGT - Reference
sgRNA
AGGACTACTACATCTCCGTGGAGTGGGACATCATGCGTGT - 71.92% (7037 reads)
AGGACTACTACATC------GAGTGGGACATCATGCGTGT - 3.45% (338 reads)
AGGACTACTACATCTC----GAGTGGGACATCATGCGTGT - 3.43% (336 reads)
AGGACTACTACATCTCCG---AGTGGGACATCATGCGTGT - 2.50% (245 reads)
AGGACTAC------------GAGTGGGACATCATGCGTGT - 1.74% (170 reads)
AGGACTACTACATCTCCGTG-AGTGGGACATCATGCGTGT - 1.50% (147 reads)
AGGACTACT-----------GAGTGGGACATCATGCGTGT - 1.38% (135 reads)
AGGACTA-------------GAGTGGGACATCATGCGTGT - 1.33% (130 reads)
AGGACTACTA----------GAGTGGGACATCATGCGTGT - 1.25% (122 reads)
AGGACTACTACA--------GAGTGGGACATCATGCGTGT - 1.11% (109 reads)
AGGACTACTACAT-------GAGTGGGACATCATGCGTGT - 1.01% (99 reads)
AGGACTACTAC---------GAGTGGGACATCATGCGTGT - 0.93% (91 reads)
AGGACT--------------GAGTGGGACATCATGCGTGT - 0.91% (89 reads)
AGGACTACTACATCTCCG--GAGTGGGACATCATGCGTGT - 0.82% (80 reads)
AGGACTACTACATCT-----GAGTGGGACATCATGCGTGT - 0.81% (79 reads)
AGGACTACTACATCTCCGTGGGAGTGGGACATCATGCGTG - 0.44% (43 reads)
AGGAC---------------GAGTGGGACATCATGCGTGT - 0.33% (32 reads)
--------------------GAGTGGGACATCATGCGTGT - 0.33% (32 reads)
AGGACTACTACATCTCCGTGTGGAGTGGGACATCATGCGT - 0.27% (26 reads)
AGGA------------------GTGGGACATCATGCGTGT - 0.21% (21 reads)
AGGACTACT----------GGAGTGGGACATCATGCGTGT - 0.21% (21 reads)
bold Substitutions
Insertions
- Deletions
------ Predicted cleavage position
